# Supplementary material for: Genome-wide association study identifies the SERPINB gene cluster as a susceptibility locus for food allergy
Source: Nat Commun. 2017 Oct 20;8:1056. doi: 10.1038/s41467-017-01220-0 (PMC5648765; doi:10.1038/s41467-017-01220-0)
Supplement: Supplementary file 1 — Description of Additional Supplementary Files [file 41467_2017_1220_MOESM1_ESM.pdf]

Description of Additional Supplementary Files

File Name: Supplementary Data 1
Description: Top associated loci (P < 1 x 10-3 in the discovery set) for any food allergy.
