# Supplementary material for: Genome-wide association study identifies the SERPINB gene cluster as a susceptibility locus for food allergy
Source: Nat Commun. 2017 Oct 20;8:1056. doi: 10.1038/s41467-017-01220-0 (PMC5648765; doi:10.1038/s41467-017-01220-0)
Supplement: Supplementary file 3 — Supplementary Information [file 41467_2017_1220_MOESM3_ESM.pdf]

Supplementary Information

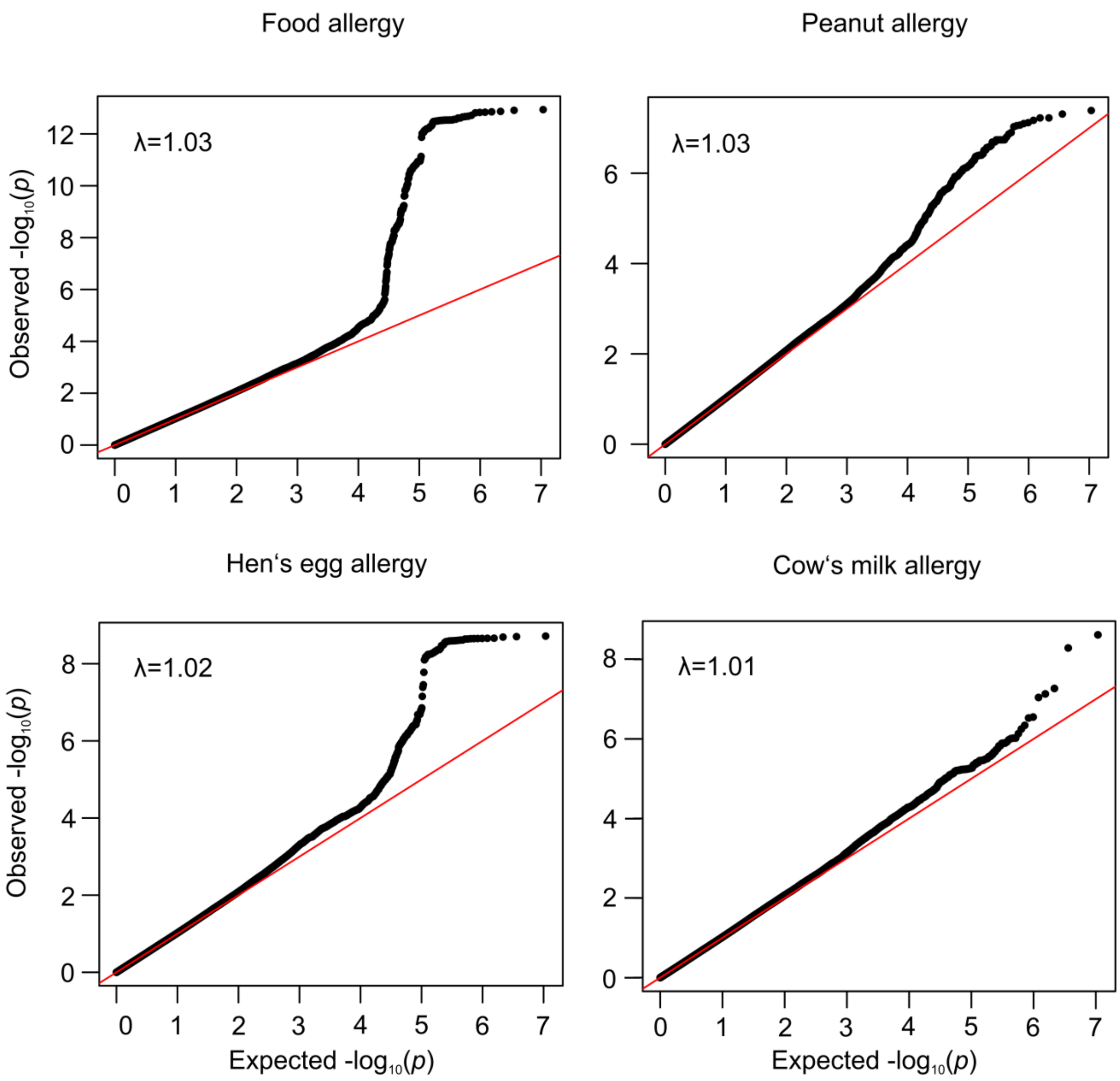

Supplementary Figure 1. Quantile-quantile plots showing the distribution of observed *P* values (x-axis) and expected *P* values (y-axis) for the GWAS performed on any food allergy and on food-specific allergies against peanut, hen's egg, and cow's milk, respectively. For each phenotype under study, the genomic inflation factor lambda is indicated.

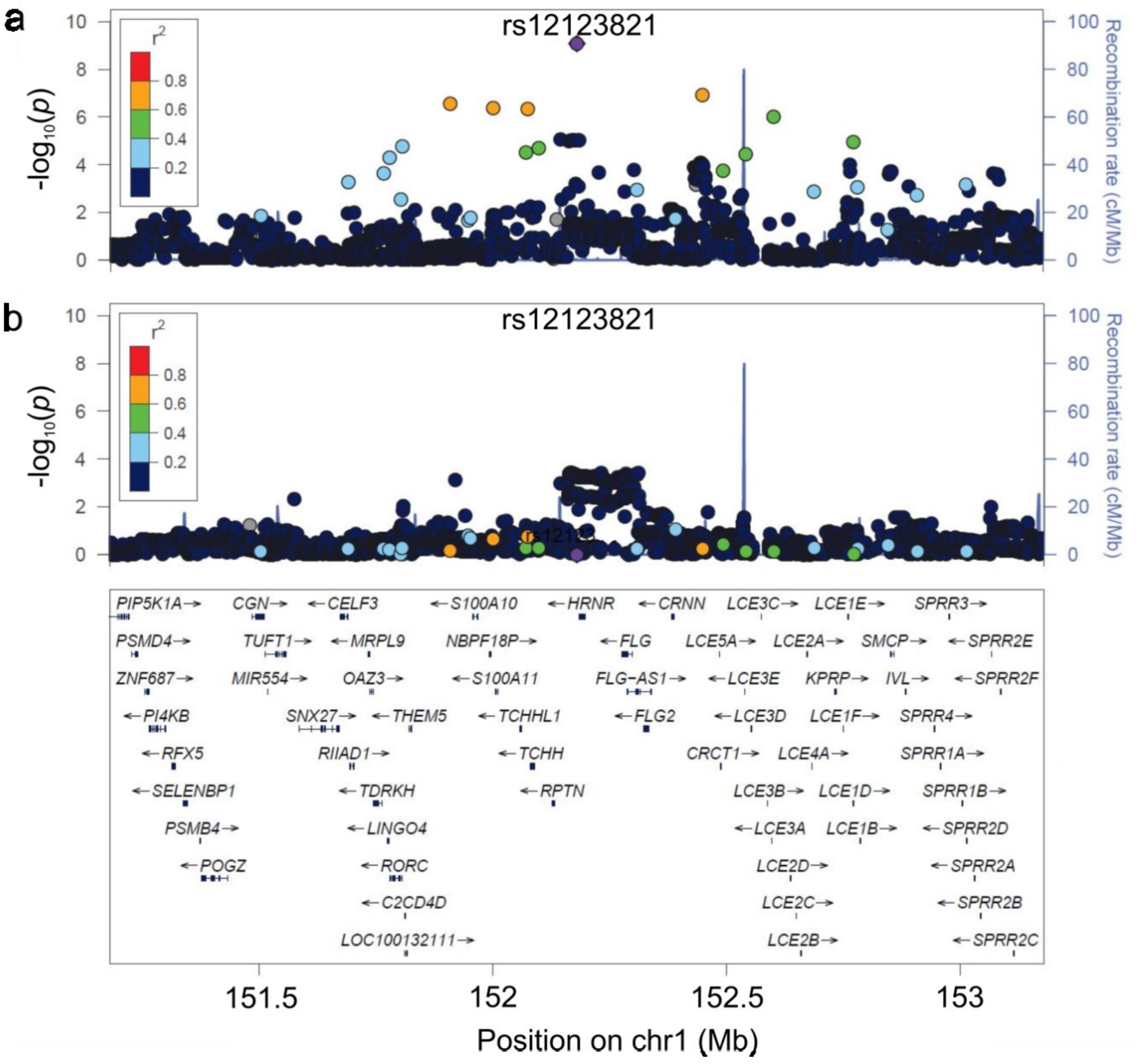

Supplementary Figure 2. Regional association plots for the filaggrin gene (*FLG*) locus at 1q21.3 before (**a**) and after (**b**) adjusting the results for the two most common loss-of-function mutations *FLG* c.2282del4 (tagged by rs12123821) and p.R501X (rs61816761). A 2-Mb window around the lead SNP rs12123821 (purple) is shown. Genomic positions (x-axis) including the annotated genes and *P* values (y-axis) of the SNPs under study (dots) for the discovery set are indicated. Dots are coloured according to the extent of LD (measured by r^2) between the respective SNP and the lead SNP in the region. Blue peaks (y-axis) represent recombination rates. Regional association plots were generated with LocusZoom (http://csg.sph.umich.edu/locuszoom/).[1]

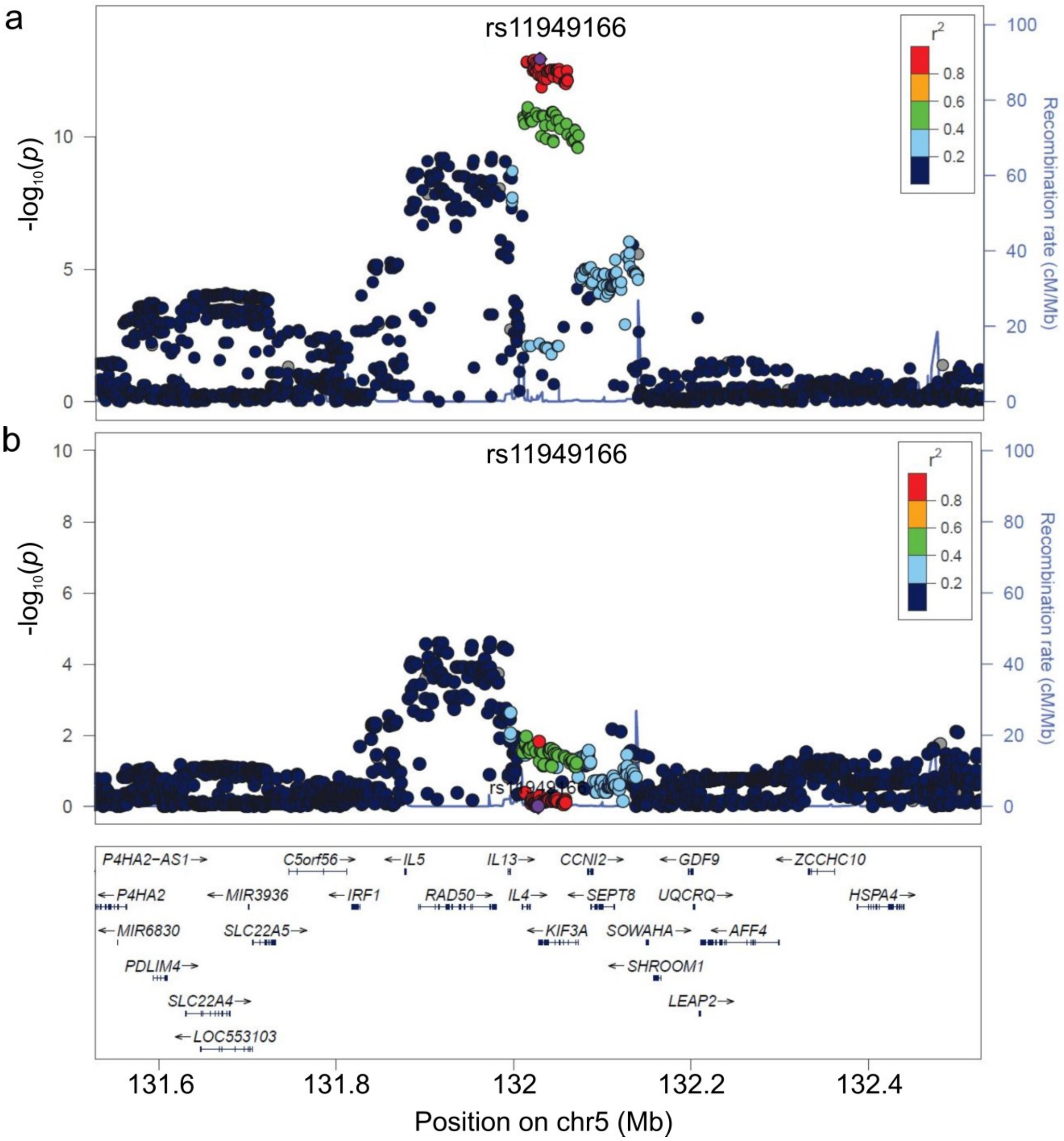

Supplementary Figure 3. Regional association plots for the cytokine gene cluster at 5q31.1 before (**a**) and after (**b**) adjusting the results for rs11949166. A 1-Mb window around the lead SNP rs11949166 (purple) is shown. Genomic positions (x-axis) including the annotated genes and *P* values (y-axis) of the SNPs under study (dots) for the discovery set are indicated. Dots are colored according to the extent of LD (measured by r^2) between the respective SNP and the lead SNP in the region. Blue peaks (y-axis) represent recombination rates.[1]

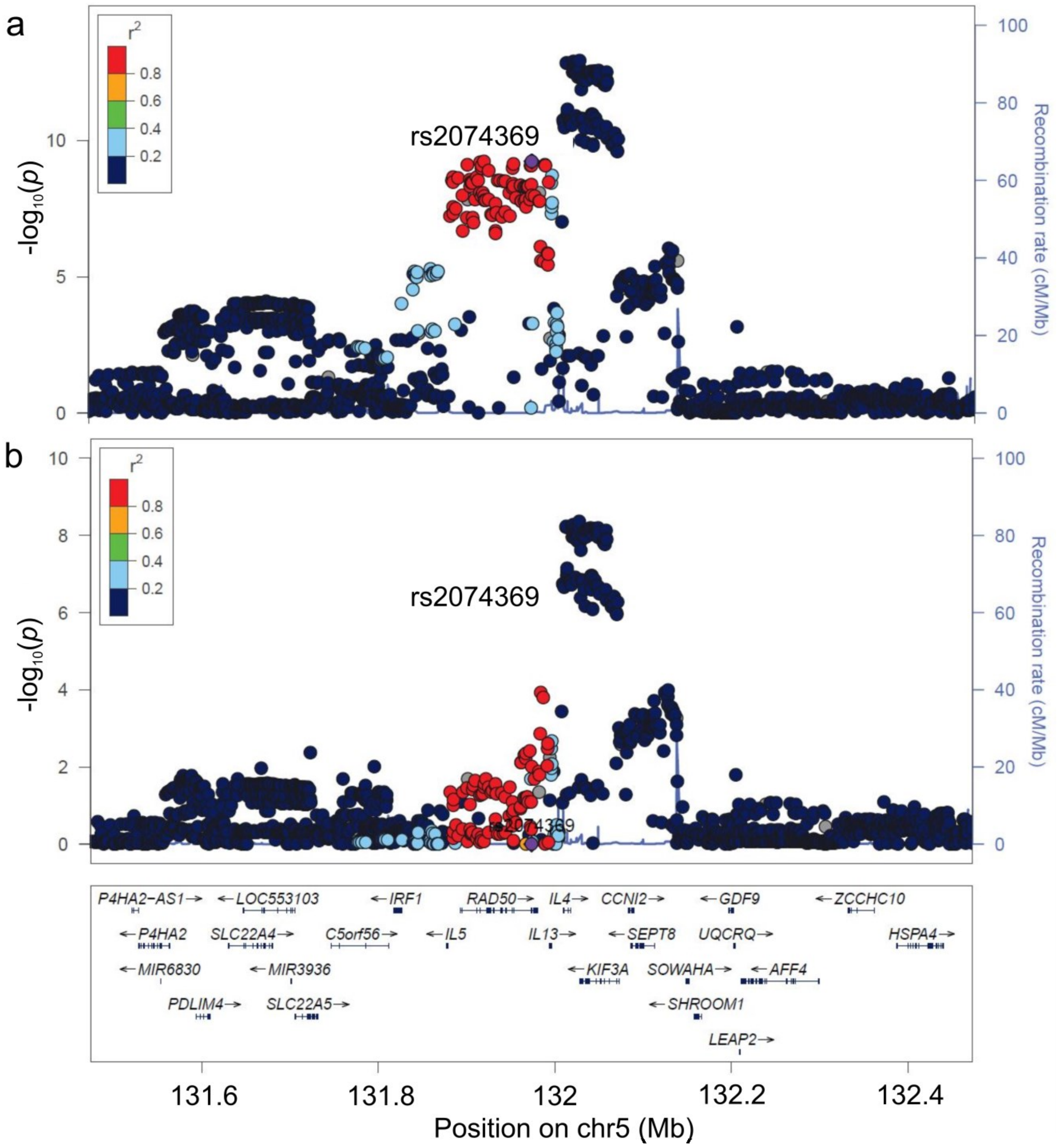

Supplementary Figure 4. Regional association plots for the cytokine gene cluster at 5q31.1 before (**a**) and after (**b**) adjusting the results for rs2074369. A 1-Mb window around the second lead SNP rs2074369 (purple) is shown. Genomic positions (x-axis) including the annotated genes and *P* values (y-axis) of the SNPs under study (dots) for the discovery set are indicated. Dots are colored according to the extent of LD (measured by r^2) between the respective SNP and the lead SNP in the region. Blue peaks (y-axis) represent recombination rates.[1]

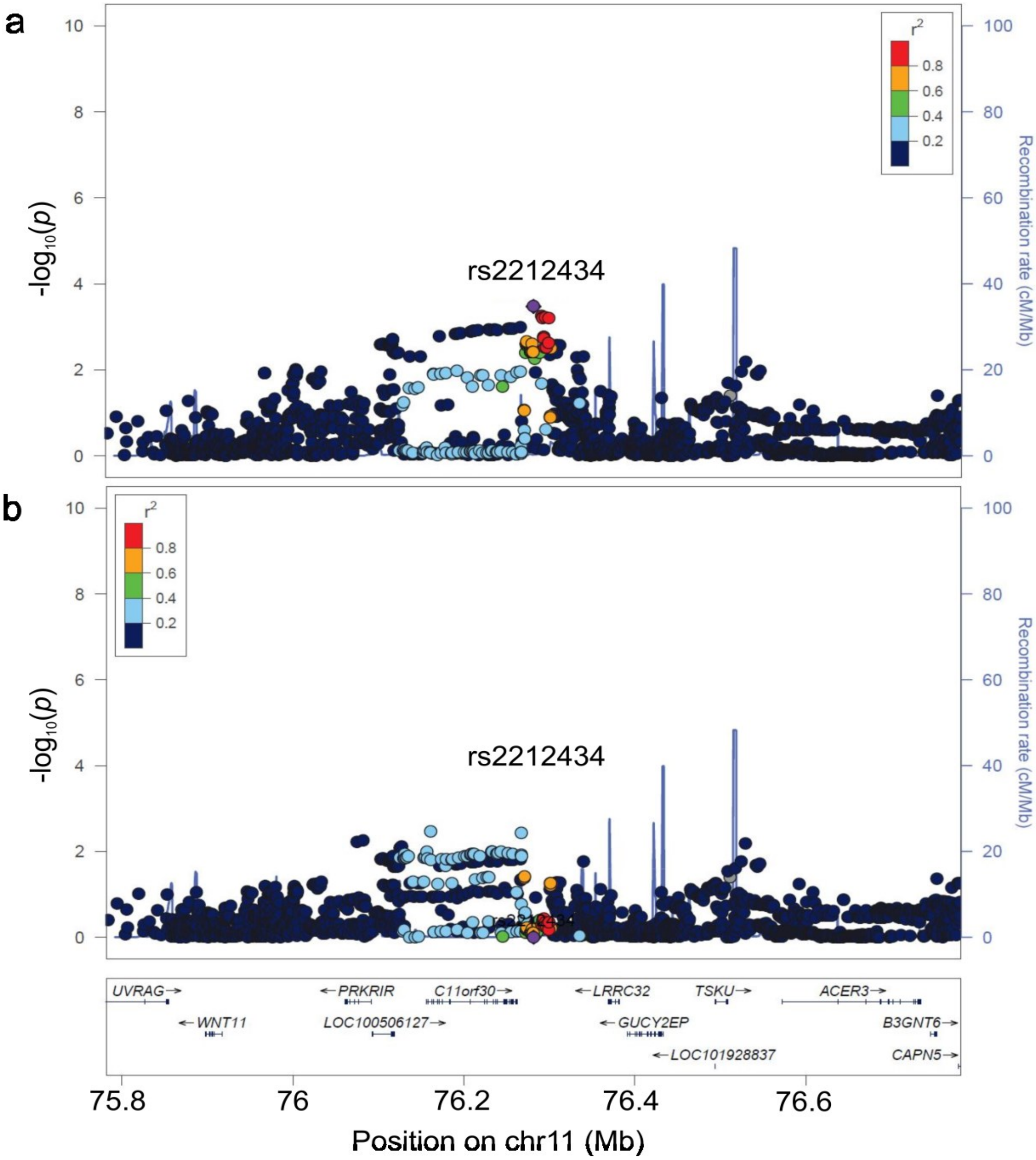

Supplementary Figure 5. Regional association plots for the food allergy locus on chromosome 11q13.5 before (**a**) and after (**b**) adjusting the results for rs2212434. A 1-Mb window around the lead SNP rs2212434 (purple) is shown. Genomic positions (x-axis) including the annotated genes and *P values* (y-axis) of the SNPs under study (dots) for the discovery set are indicated. Dots are colored according to the extent of LD (measured by r^2) between the respective SNP and the lead SNP in the region. Blue peaks (y-axis) represent recombination rates.[1]

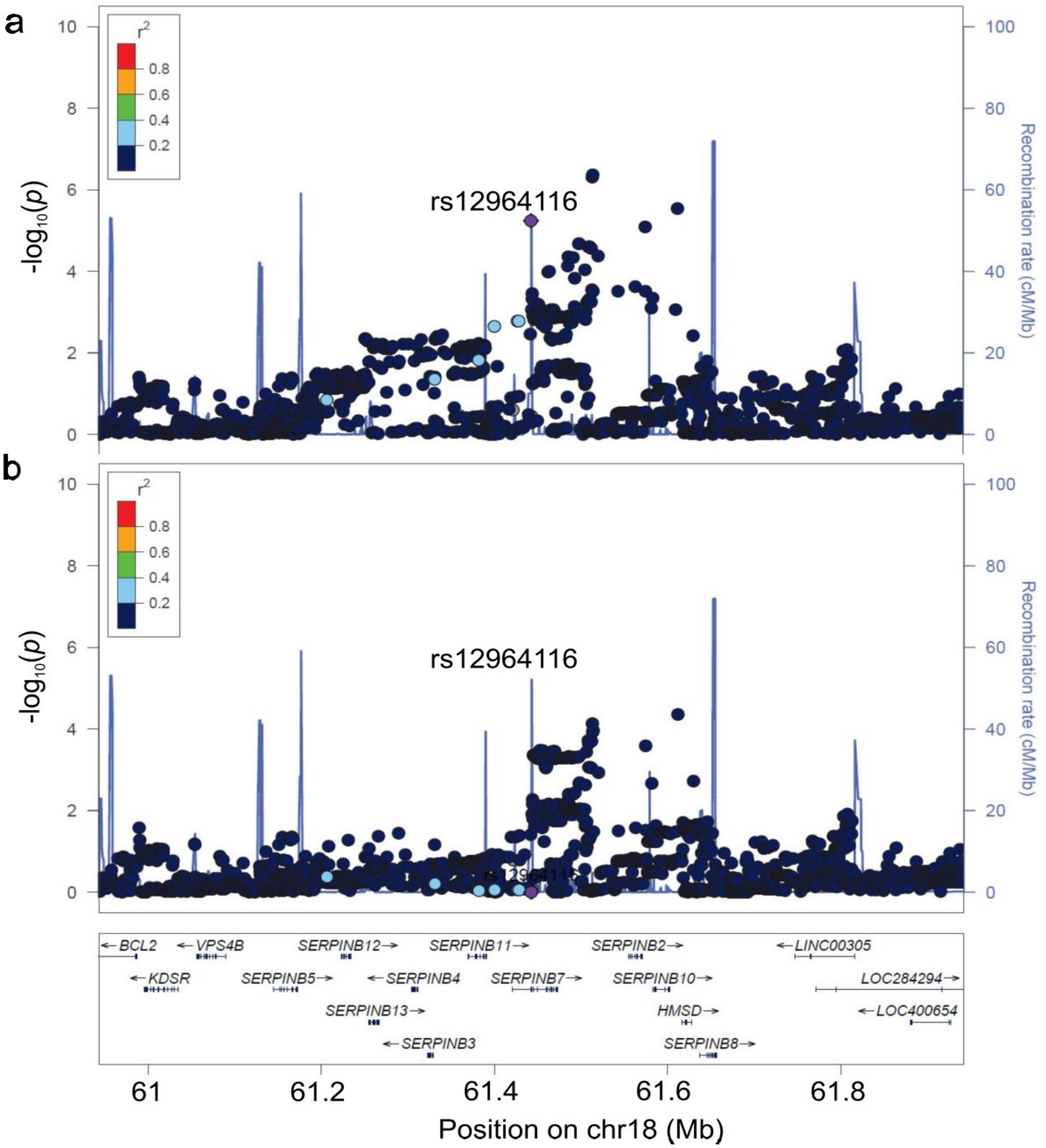

Supplementary Figure 6. Regional association plots of the *SERPINB* gene cluster at 18q21.3 before (**a**) and after (**b**) adjusting the results for rs12964116. A 1-Mb window around the lead SNP rs12964116 (purple) is shown. Genomic positions (x-axis) including the annotated genes and *P* values (y-axis) of the SNPs under study (dots) for the discovery set are indicated. Dots are coloured according to the extent of LD (measured by r^2) between the respective SNP and the lead SNP in the region. Blue peaks (y-axis) represent recombination rates.[1]

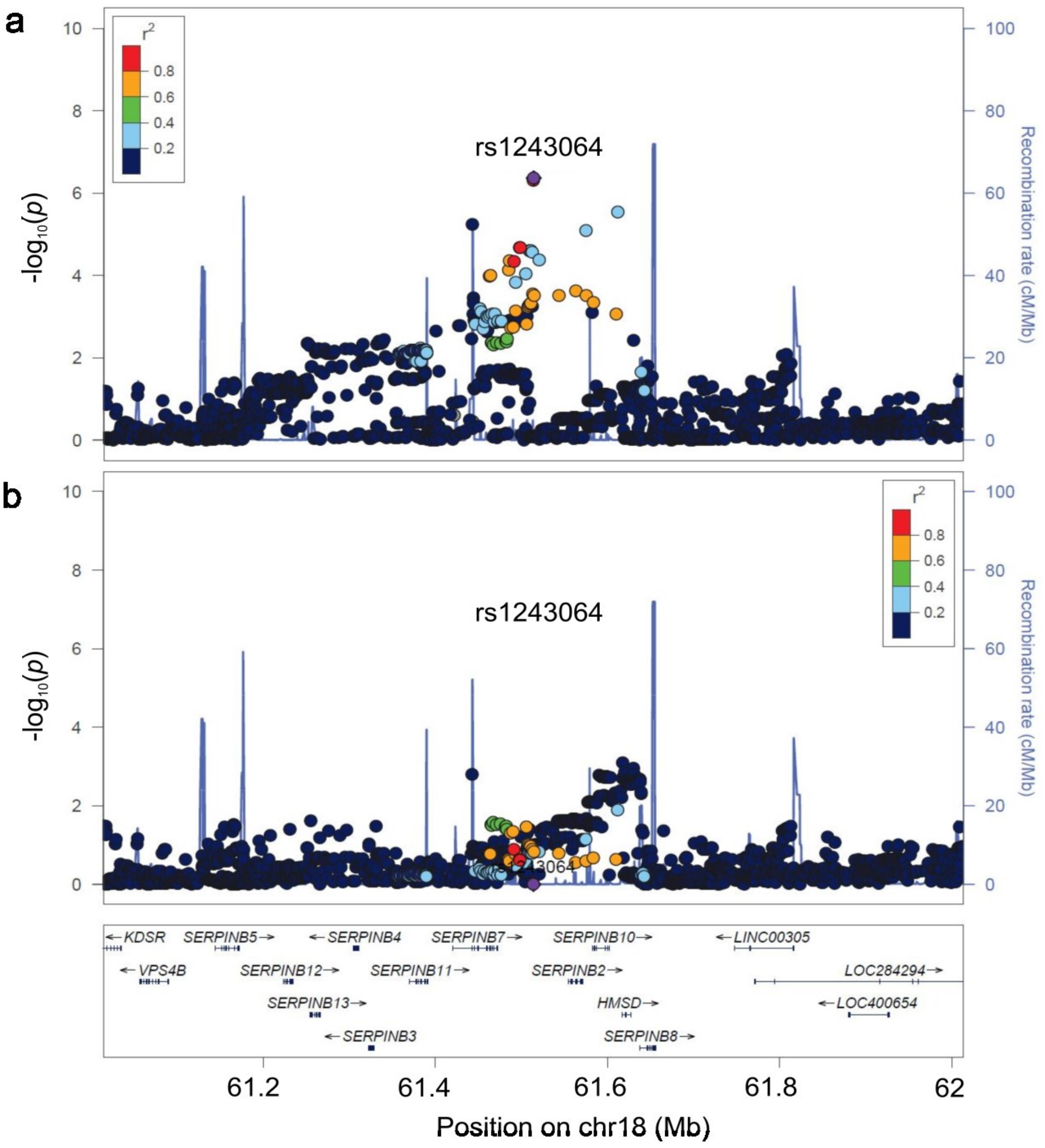

Supplementary Figure 7. Regional association plots of the *SERPINB* gene cluster at 18q21.3 before (**a**) and after (**b**) adjusting the results for rs1243064. A 1-Mb window around the second lead SNP rs1243064 (purple) is shown. Genomic positions (x-axis) including the annotated genes and *P* values (y-axis) of the SNPs under study (dots) for the discovery set are indicated. Dots are coloured according to the extent of LD (measured by r^2) between the respective SNP and the lead SNP in the region. Blue peaks (y-axis) represent recombination rates.[1]

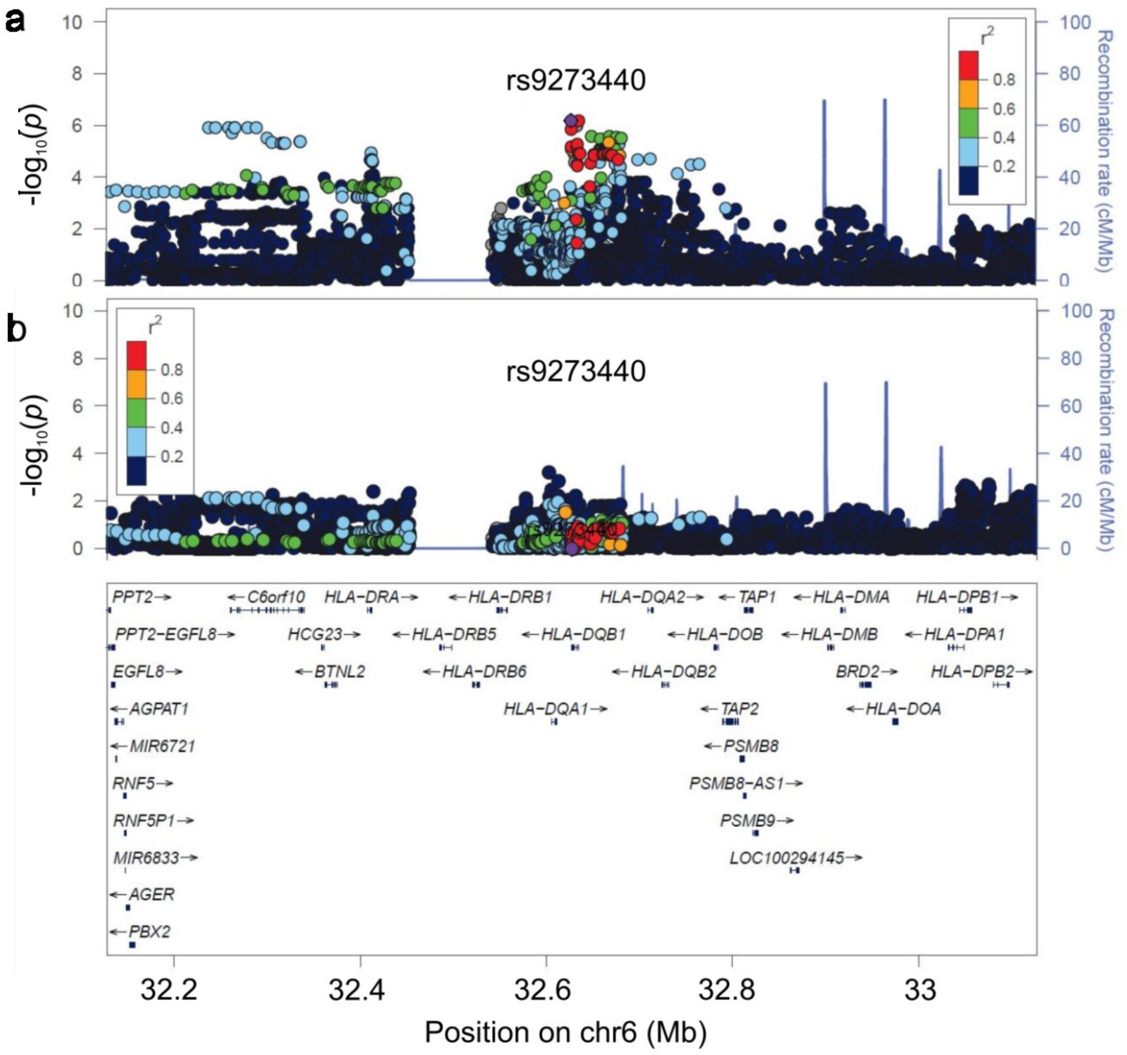

Supplementary Figure 8. Regional association plot of the HLA locus at 6p21 before (**a**) and after (**b**) adjusting for rs9273440. A 1-Mb window around the lead SNP rs9273440 (purple) is shown. Genomic positions (x-axis) including the annotated genes and *P* values (y-axis) of the SNPs under study (dots) for the discovery set are indicated. Dots are coloured according to the extent of LD (measured by r^2) between the respective SNP and the lead SNP in the region. Blue peaks (y-axis) represent recombination rates.[1]

Supplementary Table 1 SNPs analyzed in the Chicago Food Allergy Study

SNP ID	Chr	Position[a]	EA	AA	GOFA discovery set *P* value	GOFA replication set *P* value	Chicago Food Allergy Study *P* value (proxy SNP)	Direction of effect	Meta analysis *P* value[b]
Food allergy									
rs2212434	11	76281593	T	C	3.4E-04	8.2E-05	1.4E-04	+++	9.2E-11
rs12964116	18	61442619	G	A	5.7E-06	9.4E-03	0.010	+++	1.8E-08
rs521267	20	934133	C	T	4.6E-04	1.5E-04	n.a.	- -	3.4E-07
Hen's egg allergy									
rs17023017	4	96025094	G	T	6.4E-04	2.2E-04	0.37 (rs1073308. r^2 = 0.98)	+++	6.8E-07
rs1243064	18	61513975	A	T	1.6E-07	0.028	0.15	+++	8.0E-08
Peanut allergy									
rs72783152	2	36952332	T	C	5.7E-05	2.6E-03	n.a.	++	5.0E-07
rs17664036	20	21220212	C	T	4.5E-05	3.4E-04	0.24	+++	1.6E-06
Cow's milk allergy									
rs73908987	2	2595633	A	G	5.9E-05	3.0E-03	n.a.	++	6.0E-07

EA, effect allele; AA, alternative allele; n.a., no SNP data or proxy SNPs (r^2>0.8) available in the Chicago Food Allergy Study.

[a]Genomic positions were based on human genome reference assembly GRCh37.p13.

[b]Meta-analyses included all study populations with data available for the respective SNP.

Supplementary Table 2 Filaggrin mutations account for the main association signals within the epidermal differentiation complex on chromosome 1

								Conditional analysis[b]		
SNP ID	Chr	Position[a]	EA	AA	AF	OR	*P* value	OR	*P* value	Gene region
rs77426698	1	151908055	A	G	0.052	2.28	2.7×10^{-7}	0.94	0.7	*THEM4/S100A10*
rs115288876	1	152000117	A	G	0.055	2.13	4.3×10^{-7}	0.62	0.23	*S100A10/S100A11*
rs12122629	1	152074116	C	A	0.058	2.09	4.5×10^{-7}	0.63	0.18	*TCHHL1/TCHH*
rs1496044	1	152144275	A	G	0.140	0.60	9.1×10^{-6}	0.68	0.0011	*RPTN/HRNR*
rs34372395	1	152167407	G	A	0.216	0.65	9.5×10^{-6}	0.73	0.0027	*RPTN/HRNR*
rs1552994	1	152171461	G	A	0.216	0.65	9.5×10^{-6}	0.73	0.0027	*RPTN/HRNR*
rs12733173	1	152173972	T	C	0.216	0.65	9.7×10^{-6}	0.73	0.0027	*RPTN/HRNR*
rs10443207	1	152182760	C	A	0.216	0.65	9.9×10^{-6}	0.73	0.0028	*RPTN/HRNR*
rs34428306	1	152184316	C	G	0.216	0.65	9.8×10^{-6}	0.73	0.0028	*RPTN/HRNR*
rs12731336	1	152448098	G	A	0.942	0.45	1.2×10^{-7}	1.10	0.58	*CRNN/LCE5A*
rs72702813	1	152600854	T	G	0.051	2.08	9.9×10^{-7}	1.03	0.76	*LCE3A/LCE2D*

EA, effect allele; AA, alternative allele; AF, effect allele frequency; OR, odds ratio.

[a]Genomic positions were based on human genome reference assembly GRCh37.p13.

[b]Association results with food allergy conditional on rs12123821 (tagging SNP for *FLG* c.2282del4) and on rs61816761 (*FLG* p.R501X).

Supplementary Table 3 Conditional analysis for the lead SNPs in the cytokine gene cluster on chromosome 5

SNP ID	LD (r^2)[a]	Chr	Position[b]	EA	AA	AF	OR	*P* value	*P* values adjusted for rs2074369	*P* values adjusted for rs11949166	Gene
rs2074369	0.04	5	131973663	C	T	0.258	1.57	1.8×10^{-10}	n.a.	2.5×10^{-5}	*RAD50* (intron variant)
rs11949166		5	132027681	T	A	0.719	0.60	1.2×10^{-13}	4.4×10^{-9}	n.a.	*IL4/KIF3A* (intergenic variant)

EA, effect allele; AA, alternative allele; AF, effect allele frequency; OR, odds ratio; n.a., not available.

[a]LD, linkage disequilibrium between the SNPs estimated by r^2.

[b]Genomic positions were based on human genome reference assembly GRCh37.p13.

Supplementary Table 4 Allele frequencies for rs2212434, rs12964116 and rs1243064 in the different control groups of the Chicago Food Allergy Study

SNP ID	Minor allele	Trait	MAF cases	MAF in controls		
				Non-allergic (n=144)	Unknown (n=1382)	Combined
rs2212434	T	FA	0.512	0.428	0.473	0.469
		HE	0.492	0.428	0.475	0.471
		PN	0.497	0.428	0.475	0.471
		CM	0.514	0.428	0.473	0.469
rs12964116	G	FA	0.061	0.038	0.050	0.049
		HE	0.065	0.038	0.052	0.051
		PN	0.084	0.038	0.050	0.049
		CM	0.041	0.038	0.051	0.050
rs1243064	A	FA	0.265	0.271	0.264	0.265
		HE	0.288	0.271	0.262	0.263
		PN	0.279	0.271	0.264	0.265
		CM	0.244	0.271	0.261	0.262

MAF, minor allele frequency; FA, food allergy; PN, peanut allergy; HE, hen’s egg allergy; CM, cow’s milk allergy.

Supplementary Table 5 Conditional analysis for the lead SNPs in the serpin clade B gene cluster on chromosome 18

SNP ID	LD (r^2)[a]	Chr	Position[b]	EA	AA	AF	OR	*P* value	*P* values adjusted for rs12964116	*P* values adjusted for rs1243064	Gene
rs12964116	0.06	18	61442619	G	A	0.050	1.90	5.7×10^{-6}	n.a.	1.6×10^{-3}	*SERPINB7* (intron variant)
rs1243064		18	61513975	A	T	0.259	1.48	4.3×10^{-7}	1.1×10^{-4}	n.a.	*SERPINB7/B2* (intergenic_variant)

EA, effect allele; AA, alternative allele; AF, effect allele frequency; OR, odds ratio; n.a., not available.

[a]LD, linkage disequilibrium between the SNPs estimated by r^2.

[b]Genomic positions were based on human genome reference assembly GRCh37.p13.

Supplementary Table 6 Functional annotations and eQTLs for the SNPs associated with food allergy

SNP ID	MAF	Position	LD (r^2,D')[a]	Gene	Functional annotation[b]	Binding proteins[c]	eQTLs[d]	Tissue (*P* value)
Chr. 5q31.1								
**rs11949166**	**0.27**	**132,027,681**	**-**	***IL4/KIF3A***	**Intergenic**	**-**	**-**	**-**
**rs2074369**	**0.20**	**131,973,663**	**-**	***RAD50***	**Intron**	**-**	**-**	**-**
Chr. 6p21								
**rs9273440**	**0.23**	**32,627,561**	**-**	***HLA-DQB1***	**3-Prime UTR**	**-**	**-**	**-**
rs1049133	0.21	32,629,847	0.83,0.97	*HLA-DQB1*	Missense variant	-	-	-
Chr. 11q13.5								
**rs2212434**	**0.44**	**76,281,593**	**-**	***C11orf30/LRRC32***	**Intergenic**	**-**	**-**	**-**
rs61893460	0.45	76,291,154	0.96,1	*C11orf30/LRRC32*	Intergenic	CTCF (H1ESC,K562,HUVEC), Rad21 (K562),Cjun,Max,PolII(HUVEC)	-	-
rs7126418	0.45	76,292,573	0.97,1	*C11orf30/LRRC32*	Intergenic	-	-	-
rs7114362	0,49	76,293,070	0.82,1	*C11orf30/LRRC32*	Intergenic	-	-	-
rs7110818	0,45	76,292,575	0.96,1	*C11orf30/LRRC32*	Intergenic	-	-	-
rs7936070	0,47	76,293,527	0.90,1	*C11orf30/LRRC32*	Intergenic	-	-	-
rs7936312	0,47	76,293,726	0.90,1	*C11orf30/LRRC32*	Intergenic	-	-	-
rs7936323	0,47	76,293,758	0.90,1	*C11orf30/LRRC32*	Intergenic	-	-	-
rs7936434	0,47	76,293,805	0.89,1	*C11orf30/LRRC32*	Intergenic	-	-	-
rs4494327	0,49	76,294,836	0.82,1	*C11orf30/LRRC32*	Intergenic	-	-	-
rs11236791	0,45	76,295,598	0.96,1	*C11orf30/LRRC32*	Intergenic	-	-	-
rs10160518	0,49	76,296,671	0.82,1	*C11orf30/LRRC32*	Intergenic	-	-	-
rs2155219	0,49	76,299,194	0.82,1	*C11orf30/LRRC32*	Regulatory	PolII ,Cjun,Max,Cmyc (HUVEC)	-	-
rs11236797	0,45	76,299,649	0.96,1	*C11orf30/LRRC32*	Regulatory	Cjun,Max,PolII,Cmyc (HUVEC)	-	-
rs7931483	0,47	76,302,067	0.87,0.99	*C11orf30/LRRC32*	Intergenic	-	-	-

Supplementary Table 6 (continued)

SNP ID	MAF	Position	LD (r^2,D')[a]	Gene	Functional annotation[b]	Binding proteins[c]	eQTLs[d]	Tissue (*P* value)
Chr. 18q21.3								
**rs12964116**	**0,04**	**61,442,619**	**-**	***SERPINB7***	**TF binding site**	**CEBPB (HeLa-S3), CJUN (HeLa-S3), JUND (HeLa-S3), P300 (HeLa-S3), STAT3 (MCF10A-Er-Src), POL2B (NHEK)**	**-**	**-**
rs71353401	0,04	61,412,756	0.82,1	*SERPINB11/SERPINB7*	Intergenic	-	-	-
**rs1243064**	**0,26**	**61,513,975**	**-**	***SERPINB7/SERPINB2***	**Intergenic**	**-**	***SERPINB10***	**Monocytes ($2.0×10^{-28}$)[2]**
							SERPINB10	**Blood ($2.9×10^{-13}$)[3]**
							SERPINB10	**Blood ($1.3×10^{-14}$)[4]**
rs1243063	0,26	61,513,490	0.98,1.0	*SERPINB7/SERPINB2*	Intergenic	-	-	-
rs986982	0,27	61,497,804	0.94,0.84	*SERPINB7/SERPINB2*	Intergenic	-	*SERPINB10*	Blood ($4.6×10^{-9}$)[5]
rs1243039	0,27	61,498,396	0.94,0.84	*SERPINB7/SERPINB2*	Intergenic	-	-	-
rs1720910	0,27	61,491,321	0.94,0.83	*SERPINB7/SERPINB2*	Intergenic	CTCF(NHEK,GM12878,Monocytes,HeLa-S3,HMEC,K562,H1ESC,HUVEC), RAD21 (H1ESC,GM12878)	-	-

MAF, minor allele frequency; LD, linkage disequilibrium. Lead SNPs are indicated in bold.

[a] SNPs in LD were identified with LDlink3.0[6] which uses phase 3 data of the 1000 Genomes Project to calculate population-specific measures of linkage disequilibrium.

[b] Functional annotations according to Ensembl Variant Effect Predictor GRCh37, release 88.[7]

[c] Binding proteins were identified in ChIP-Seq experiments in the indicated cell lines.[8]

[d] eQTL data were derived from the Zeller *et al.*[2], Grundberg *et al.*[3], Westra *et al.*[4] and GTEx[5] studies accessed through the SNIPA webpage at http://www.snipa.org (SNIPAv3.2, accessed 04/2017).[9]

Supplementary Table 7 SNP-based heritability for food allergy estimated with LD score regression

Prevalence used in LD-score regression		Heritability estimate (A, all SNPs)		Heritability estimate (B, without lead SNPs)		h^2 explained by the identified lead SNPs	
Population	Sample	h^2	SE	h^2	SE	Absolute (A – B)	Relative to overall SNP heritability (A – B) / A
0.05	0.26	0.244	0.146	0.219	0.132	0.025	0.102

h^2, heritability on the liability-scale; SE, standard error.

Supplementary Table 8 Tissue-specific gene expression within the serpin clade B gene cluster at 18q21.3

Gene	Adrenal gland	Aorta	Blood	Breast	Cerebral cortex	Cortex of kidney	EBV-transformed lymphocyte	Esophagus mucosa	Heart left ventricle	Liver	Lung	Minor salivary gland	Ovary	Pancreas	Prostate gland	Sigmoid colon	Skeletal muscle tissue	Small intestine	Spleen	Stomach	Suprapubic skin	Testis	Thyroid gland	Uterus	Vagina
SERPINB5	0.1			2	0.1		0.1	68			0.1	6	0.1	0.1	2	0.1		4	0.1	0.1	17	1	0.1	0.1	34
SERPINB12								1													2	0.2			3
SERPINB13								82				0.1			0.1						6				69
SERPINB4								2				0.1			0.1						0.2	0.2			33
SERPINB3								131				0.2			0.3						3	0.7			129
SERPINB11								4				0.1			3							0.1			3
SERPINB7						0.1		0.5				0.1			0.1						48	0.1			0.7
SERPINB2		0.1	1	0.1			0.1	42			0.2	0.5		0.1					0.1		18				9
SERPINB10			1				3	0.2			0.1								0.2		0.2				0.1
HMSD[b]		0.1			0.3		6	0.1		0.1	0.4				0.1	0.1		0.1	0.2		0.1	0.2	0.1		0.1
SERPINB8	3	4	3	3	0.6	1	4	7	0.6	1	4	3	2	0.9	2	1	0.5	3	3	2	11	0.8	2	1	4

[a] Expression data are from the latest release of the GTEx project (V6p)[5] and were accessed through the Expression Atlas webpage.[10] Expression values are shown in RPKM (Reads Per Kilobase of transcript per Million mapped reads), calculated from a gene model with isoforms collapsed to a single gene.[5] A representative selection of human tissues is shown.

[b] Histocompatibility minor serpin domain containing gene.

Supplementary Table 9 Genotyping and imputation platforms used in this study

	GOFA discovery set	GOFA replication set	Chicago Food Allergy Study
Genotyping array (no. of markers)	Illumina HumanOmniExpress Exome-8 v1.2 (980,000)	Illumina HumanOmniExpress Exome-8 v1.2 (980,000)	Illumina HumanOmni1 Quad (1.1 M)
	Illumina HumanOmniExpress 12 v1.0 (730,000)	Illumina HumanOmni2.5 Quad (2.3 M)	
	Illumina HumanOmni1 Quad v1.0 (1.1 M)		
Genotyped markers after QC	606,699 (autosomal)	625,579 (autosomal)	789,677
Imputation / phasing	SHAPEITv2 / minimac3	SHAPEITv2 / minimac3	SHAPEIT / IMPUTE2
Reference panel	Haplotype Reference Consortium (version1.1)	Haplotype Reference Consortium (version1.1)	1000 Genomes (phase I, release_v3)
Imputed markers after QC	5,405,832	5,412,934	6,459,842

QC, quality control.

Supplementary Note 1 Age of eczema onset in children with food allergy

Age of eczema onset was assessed in the GOFA study sets. The mean age at food allergy diagnosis was 2.1 and 2.8 years in the GOFA discovery and GOFA replication sets. However, follow-up data was available in a large number of cases, the mean age at last follow-up was 69 months. Of 717 GOFA cases with eczema, 590 (82.3 %) had age of eczema onset available, including 381 children aged 0-5 years, 140 children aged 6-10 years and 69 children >10 years. The mean age of eczema onset was 4.5 months (95% CI, 3.9-4.6 months) among 0-5 year olds, 4.6 months (95% CI, 4.0-5.3 months) among 5-10 year olds, and 7.2 months (95% CI, 4.1-10.2 months) among >10 year olds. The maximum age of onset observed in children >10 years was 48 months.

Although eczema may develop in later childhood or adulthood, this seems to be a rare event in children with food allergy. Of 69 children with follow-up >10 years of age (mean 13.1 years, 95% CI, 12.6-13.7 years), 92.7% manifested eczema within the first year of life, and 95.7% within the first 2 years of life. New onset eczema was not observed after the age of 48 months. The mean age at last follow-up in GOFA children without eczema was 81 months. It is therefore unlikely that the proportion of eczema among food allergic children was underestimated.

Supplementary References

1. Pruim RJ, *et al.* LocusZoom: regional visualization of genome-wide association scan results. *Bioinformatics* **26**, 2336-2337 (2010).

2. Zeller T, *et al.* Genetics and beyond--the transcriptome of human monocytes and disease susceptibility. *PLoSONE* **5**, e10693 (2010).

3. Grundberg E, *et al.* Mapping cis- and trans-regulatory effects across multiple tissues in twins. *NatGenet* **44**, 1084-1089 (2012).

4. Westra HJ, *et al.* Systematic identification of trans eQTLs as putative drivers of known disease associations. *NatGenet* **45**, 1238-1243 (2013).

5. GTEx Consortium. Human genomics. The Genotype-Tissue Expression (GTEx) pilot analysis: multitissue gene regulation in humans. *Science* **348**, 648-660 (2015).

6. Machiela MJ, Chanock SJ. LDlink: a web-based application for exploring population-specific haplotype structure and linking correlated alleles of possible functional variants. *Bioinformatics* **31**, 3555-3557 (2015).

7. McLaren W, *et al.* The Ensembl Variant Effect Predictor. *Genome Biol* **17**, 122 (2016).

8. Goi C, Little P, Xie C. Cell-type and transcription factor specific enrichment of transcriptional cofactor motifs in ENCODE ChIP-seq data. *BMCGenomics* **14 Suppl 5**, S2 (2013).

9. Arnold M, Raffler J, Pfeufer A, Suhre K, Kastenmuller G. SNiPA: an interactive, genetic variant-centered annotation browser. *Bioinformatics* **31**, 1334-1336 (2015).

10. Petryszak R, *et al.* Expression Atlas update--an integrated database of gene and protein expression in humans, animals and plants. *Nucleic Acids Res* **44**, D746-D752 (2016).
